# Supplementary material for: Toxicological Effects of Titanium Dioxide Nanoparticles on Human Menstrual Blood Mesenchymal Stem Cells
Source: Int J Mol Sci. 2025 Nov 19;26(22):11168. doi: 10.3390/ijms262211168 (PMC12652598; doi:10.3390/ijms262211168)
Supplement: Supplementary file 1 [file ijms-26-11168-s001.zip › ijms-3804280-supplementary.pdf]

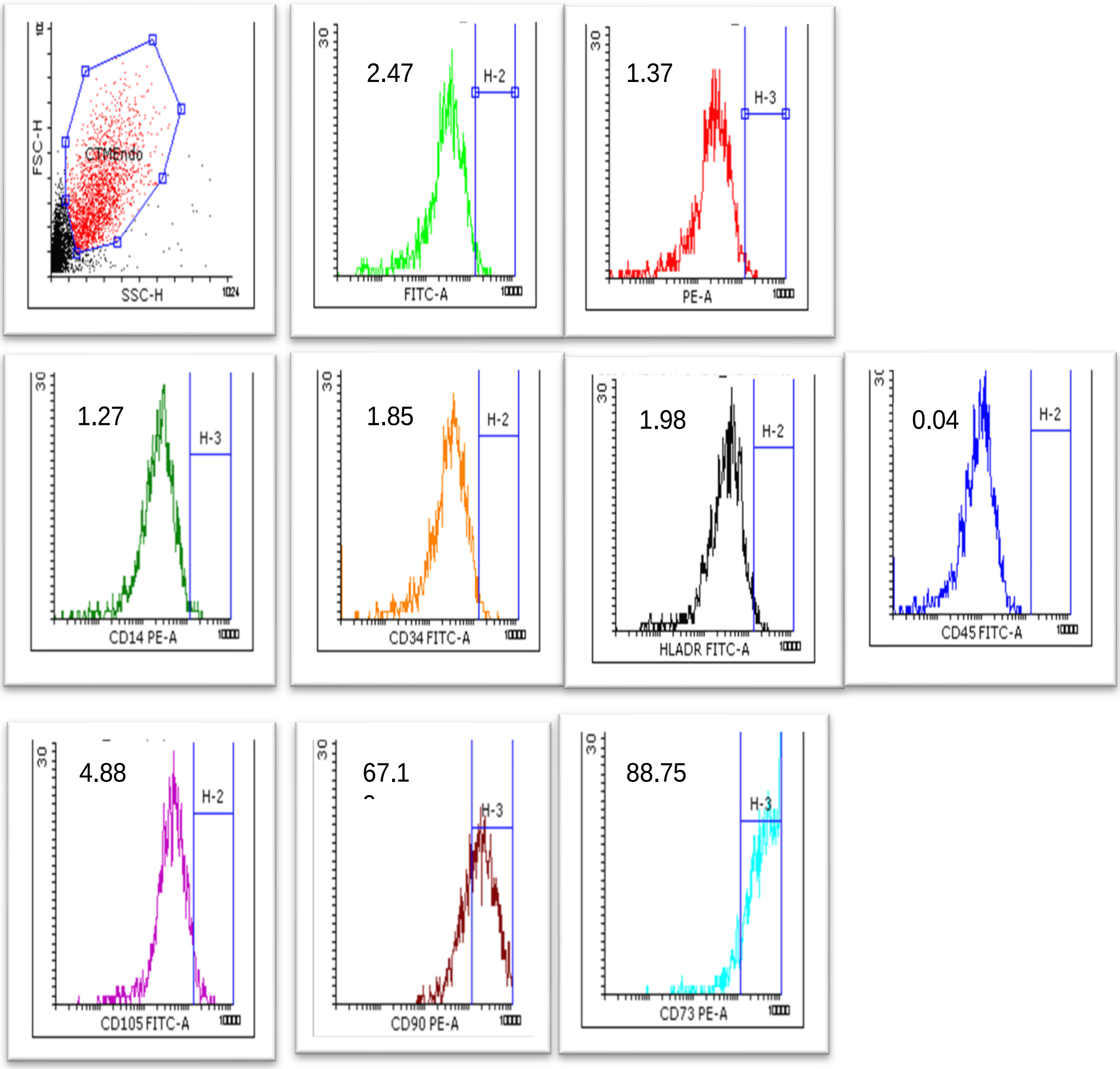

Figure S1. Flow cytometry analysis was conducted to evaluate the expression of various membrane markers. The results indicated that the markers for non-hematopoietic cells (CD73, CD90, and CD105) were positive, while those for hematopoietic cells (CD14, CD34, CD45, and HLA-DR) were negative. (n = 3).

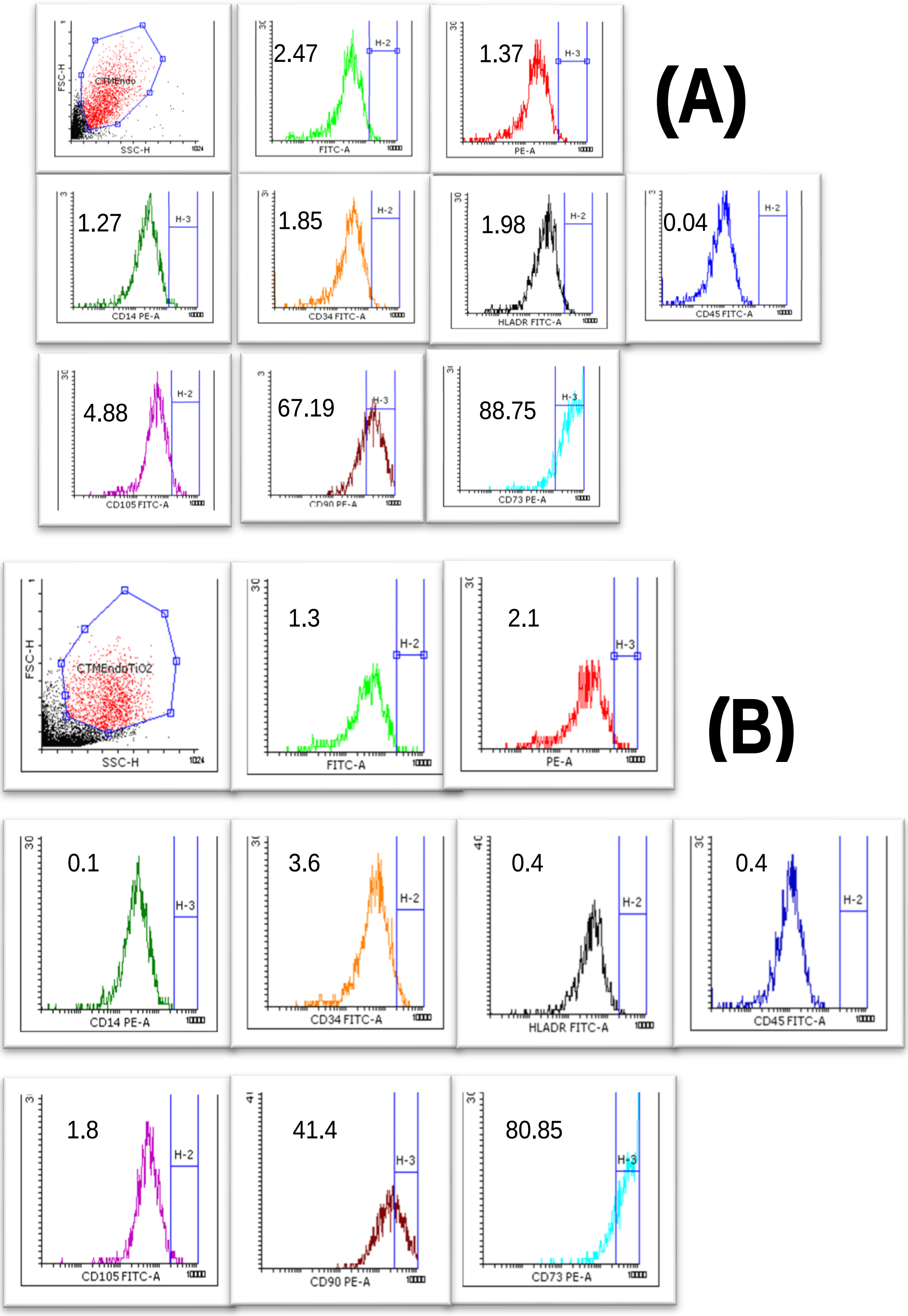

Figure S2. TiO_2 NPs attenuate the expression of surface markers in hMB-MSC. A dot blot analysis of the cell population was performed, selecting the R1 region (upper) for histograms depicting autofluorescence in both treated and control cells (with FITC represented by the green line and PE by the red line). (**A**) This illustrates hMB-MSC in the untreated condition. (**B**) This shows hMB-MSC that were exposed to 62.5 µg/mL of TiO_2 NPs for 24 h (lower histograms). Subsequently, the cells were stained with corresponding monoclonal antibodies to assess the expression of specific molecules on their surface. The representative images are derived from three independent assays (n = 3). FITC refers to fluorescein isothiocyanate, while PE denotes phycoerythrin.

Supplemental Materials Letter S1. Informed Consent Form for Endometrial Tissue Donation

Registration Number: 001-02-2023ENDO

Donor's Name: ____ Date: _____

______________________________ Age:_______________________

Health Status: Healthy woman, no history of chronic degenerative diseases, negative serological tests for blood-transmissible diseases.

Purpose of the Study

You are being invited to voluntarily donate a sample of your endometrial tissue, obtained menstrual blood. The purpose of collecting this tissue is to isolate mesenchymal stem cells (MSCs), which will be used exclusively for basic, preclinical, and clinical research protocols. These studies aim to advance scientific knowledge and develop potential therapeutic applications.

Procedure

The menstrual blood procedure will be performed by qualified medical professionals under sterile conditions. The menstrual blood sample was collected on first two days of menstruation. The procedure may cause temporary discomfort and the risks are minimal.

Risks and Discomforts

1. Temporary discomfort by used the vaginal cup
2. Rare risk of infection, which will be minimized by sterile technique

There are no long-term health risks associated with this procedure.

Benefits

There is no direct medical benefit to you from this donation. However, your participation will contribute to important research that may benefit future patients.

Confidentiality and Data Protection

3. Your personal information will be kept strictly confidential.

4. All samples will be coded with the registration number provided above; your identity will not appear in any publications or shared data.

5. Data will be handled according to international data protection laws and ethical guidelines (including GDPR and equivalent regulations).

Voluntary Participation and Right to WithdrawParticipation in this study is completely voluntary.

6. You may withdraw your consent at any time without any negative consequences.
7. If you withdraw, your sample and related data will be securely destroyed.

Ethical Considerations

This study follows the principles of the Declaration of Helsinki, Council for International Organizations of Medical Sciences (CIOMS) guidelines, and Good Clinical Practice (ICH-GCP) standards.

Contacts for Questions

If you have any questions regarding the procedure, risks, or your rights as a participant, please contact the research team at: Corporativo Exomelab / Contact: 52 55 13898166.

Consent Statement

I, ____, have read and understood the information provided above. I voluntarily agree to donate my endometrial tissue for the purposes described. I understand that my identity will remain confidential and that I may withdraw my consent at any time.

Donor's Name:____

Date: 02/02/2023

Investigator's Name: Biol. Ricardo Rangel Martínez

Date: 02/02/2023

Table S1. Zeta potencial and electrophoretic mobility values of TiO_2 NPs suspended in different solutions.

SAMPLE	ζ Zeta Potential (mV)	Electrophoretic Mobility (μmcm/Vs)
TiO_2 NPs sterile suspended in medium + serum	−6.98	−0.547
TiO_2 NPs non sterile suspended in medium + serum	−7.57	−0.593
TiO_2 NPs sterile suspended in medium free serum	−19.5	−1.532
TiO_2 NPs non sterile suspended in medium free serum	−14.6	−1.141

The values were obtained in a Zetasizer Nano series model ZS.
